# Patient- and delivery-level factors related to acceptance of HIV counseling and testing services among tuberculosis patients in South Africa: a qualitative study with community health workers and program managers

**DOI:** 10.1186/1748-5908-6-27

**Published:** 2011-03-23

**Authors:** J Christo Heunis, Edwin Wouters, Wynne E Norton, Michelle C Engelbrecht, N Gladys Kigozi, Anjali Sharma, Camille Ragin

**Affiliations:** 1 Centre for Health Systems Research & Development, University of the Free State, (205 Nelson Mandela Drive), Bloemfontein, (9300), South Africa; 2 Department of Sociology and Research Centre for Longitudinal and Life Course Studies, University of Antwerp, (2 Sint Jacob Street), Antwerp, (2000), Belgium; 3 Department of Health Behavior, School of Public Health, University of Alabama at Birmingham, (1665 University Boulevard), Birmingham, Alabama, (35294-0022),USA; 4 Division of Infectious Diseases, State University of New York, Downstate Medical Center, (450 Clarkson Avenue), Brooklyn, New York, (11203), USA; 5 Department of Epidemiology, State University of New York, Downstate Medical Center, (450 Clarkson Avenue), Brooklyn, New York, (11203), USA

## Abstract

**Background:**

South Africa has a high tuberculosis (TB)-human immunodeficiency virus (HIV) coinfection rate of 73%, yet only 46% of TB patients are tested for HIV. To date, relatively little work has focused on understanding why TB patients may not accept effective services or participate in programs that are readily available in healthcare delivery systems. The objective of the study was to explore barriers to and facilitators of participation in HIV counseling and testing (HCT) among TB patients in the Free State Province, from the perspective of community health workers and program managers who offer services to patients on a daily basis. These two provider groups are positioned to alter the delivery of HCT services in order to improve patient participation and, ultimately, health outcomes.

**Methods:**

Group discussions and semistructured interviews were conducted with 40 lay counselors, 57 directly observed therapy (DOT) supporters, and 13 TB and HIV/acquired immune deficiency syndrome (AIDS) program managers in the Free State Province between September 2007 and March 2008. Sessions were audio-recorded, transcribed, and thematically analyzed.

**Results:**

The themes emerging from the focus group discussions and interviews included four main suggested barrier factors: (1) fears of HIV/AIDS, TB-HIV coinfection, death, and stigma; (2) perceived lack of confidentiality of HIV test results; (3) staff shortages and high workload; and (4) poor infrastructure to encourage, monitor, and deliver HCT. The four main facilitating factors emerging from the group and individual interviews were (1) encouragement and motivation by health workers, (2) alleviation of health worker shortages, (3) improved HCT training of professional and lay health workers, and (4) community outreach activities.

**Conclusions:**

Our findings provide insight into the relatively low acceptance rate of HCT services among TB patients from the perspective of two healthcare workforce groups that play an integral role in the delivery of effective health services and programs. Community health workers and program managers emphasized several patient- and delivery-level factors influencing acceptance of HCT services.

## Background

In South Africa, approximately 73% of tuberculosis (TB) patients are coinfected with human immunodeficiency virus (HIV) [1]. Although integrated treatment and care is critical for improving the health of TB-HIV coinfected patients, as well as reducing transmission of both diseases to uninfected others, less than half (46%) of TB patients accept HIV counseling and testing (HCT) in the Free State Province [2].

Despite the clear need for the implementation of HCT in TB care settings, numerous barriers at the patient and provider levels exist that account in part for relatively low receipt of HCT among TB patients. Our previous research [3] in the Free State Province suggests that TB patients are reluctant to request or receive HCT when they had not received information on the relationship between TB and HIV at the health facility and when they are male, married, employed, undergoing first rather than retreatment for TB, and when they do not know someone with or have not lost someone due to HIV/acquired immune deficiency syndrome (AIDS). Internationally, a wide range of patient-level factors have been variably associated with TB patients' nonuptake of HIV testing, including female sex [4-7], age younger than 15 and older than 49 years [7], age younger than 30 and older than 39 years [4,5], age older than 46 years [6], age older than 18 years [8], fear of stigmatization [9-11], and fears of testing HIV-positive and death [11,12].

Previously identified provider-level barriers to implementing HCT with TB patients in sub-Saharan Africa [13] and South Africa [14] include lack of nursing staff, lack of space, increased workload, and work-related stress, including stress experienced by breaking bad news and handling ethical dilemmas. A Ugandan study [15] identified a range of additional health-systems factors affecting the implementation of collaborative TB-HIV services, including poor TB-HIV planning, coordination, and leadership; inadequate dissemination of policy; inadequate provider knowledge; limited TB-HIV interclinic referral; poor service integration and recording; logistical shortages; and high costs of services. Another South African study [16] identifying constraints to integrating TB and HIV care in primary healthcare clinics singled out high service loads at both the TB and HIV entry points, duplication of services and underutilization of staff, and TB and HIV services functioning independently of each other.

However, relatively little research has sought to identify and understand barriers and facilitators to TB patients' participation in HCT from the perspective of community health workers (*i.e*., lay HIV counselors and directly observed treatment [DOT] supporters) and TB and HIV/AIDS program managers. Such information is especially important in resource-limited settings where both community health workers and program managers are an integral part of the healthcare delivery system. Each of these groups represents different levers for change for potentially increasing TB patients' participation in HCT services by improving or altering the implementation of such services in clinical and community care settings.

Compared to other healthcare providers, community health workers are uniquely positioned to understand and influence patients' behaviors, as well as to improve the delivery of effective health services and programs to enhance patient health. Indeed, experience in Haiti showed that community health workers had an important role in being able to enhance community uptake of services and target vulnerable groups [17]. Given their growing presence, multi-skilling, and importance in public health systems [18-23], community health workers may be uniquely situated to influence patients' behavior-including acceptance of HIV testing. However, despite their unique position to impact patient behavior as well as improve health service delivery, community health workers are rarely consulted for their professional opinion [24]. This is also the case in the Free State Province, South Africa.

While community health workers have an influential role with patients and in the delivery mechanism for providing HCT services, TB and HIV/AIDS program managers are uniquely positioned to affect policy to improve the implementation and delivery of effective healthcare programs and care. Generally speaking, program managers are responsible for developing and maintaining successful TB and HIV/AIDS control programs, in addition to securing financial and organizational support for continuous and uninterrupted supply of treatment. Despite their influential position, program managers' perceptions on HCT for TB patients have also received little attention in the literature to date. In one study, three district disease-control managers in Indonesia were interviewed on barriers to introducing HIV testing among TB patients [25]. Managers perceived poor patient-provider communication as one of the most influential barriers to acceptance of voluntary counseling and testing (VCT) among TB patients.

Based on the lack of qualitative work in this area and within this particular setting, the objective of the present study was to explore community health workers' and TB and HIV/AIDS program managers' perspectives on barriers to and facilitators of acceptance of HCT services among TB patients in Free State Province, South Africa. This article follows on two previous reports in the same setting and time period, one on predictors of TB patients' acceptance of HCT [3] and one on primary healthcare nurses' [14] perspectives on acceptance of HCT services among TB patients. Importantly, we examined not only community health workers' and program managers' perceptions of patient-level barriers and facilitators to acceptance of HCT services among TB patients, but also their perceptions about how the delivery of HCT services might improve acceptance rates. The research study was approved by the Free State Department of Health and the Committee for Research Ethics, Faculty of the Humanities, University of the Free State.

## Methods

### Setting

The current study employed qualitative research methods (*i.e*., focus group discussions and semistructured interviews) to better understand the perspective of community health workers and program managers on factors influencing HCT acceptance among TB patients. A series of group discussions and interviews were conducted with lay counselors, DOT supporters, and program managers between September 2007 and March 2008. Except for the interviews with national and provincial program managers, information was gathered from community health workers and program managers in two districts (*i.e*., Thabo Mofutsanyana and Lejweleputswa) in Free State Province, South Africa. In an effort to reflect the mix of urban/large town and rural/small town subdistricts in both the Thabo Mofutsanyana and Lejweleputswa districts, participants in each district were recruited from a variety of purposefully selected clinics and district and regional hospitals across the two districts. A total of 19 healthcare delivery facilities were selected for participation in the present study (Table 1). These included 13 primary healthcare clinics, 5 district hospitals, and 1 regional hospital. A heterogeneous mix of facilities was selected to provide a representative set of findings.

**Table 1:** Sampled facilities types by category of community health worker

Facility type	Lejweleputswa District		Thabo Mofutsanyana District	
	
	Lay counselors	DOT supporters	Lay counselors	DOT supporters
PHC facility	8	27	15	30

District hospital	8	0	6	0

Regional hospital	3	0	0	0

**Total**	19	27	21	30

DOT = directly observed therapy; PHC = primary healthcare

### Participants

Participation in the study was voluntary, and, all being literate, participants provided written informed consent. Different recruitment strategies were used for the groups of respondents who participated in the study.

### Lay counselors

Exploratory group interviews were conducted with 40 lay counselors. All lay counselors at the selected facilities were approached to participate in the study via their supervisors and all agreed to take part.

### DOT supporters

Exploratory group interviews were conducted with 57 DOT supporters at the selected facilities. Supervisors informed all DOT supporters about the research and invited them to participate in the study. Again, all agreed to be interviewed.

### Program managers

Exploratory individual interviews were conducted with 13 TB and HIV/AIDS program managers. Unlike with the community health workers, it was not feasible to gather program managers for group interviews. Also, because the managers formed part of a hierarchy of positions subordinate to one another, and thus information could be biased by the power exerted by some over others, the group interview was not an appropriate approach for data collection. Hence, the strategy was to conduct individual interviews with the managers. The selected managers (subdistrict, n = 3; district, n = 2; provincial, n = 4; national, n = 4) represented a purposive sample to cover program managers at all levels of the public health system. Due to our undertaking to protect the confidentiality of the respondents, further details on their location and specific portfolios are withheld. Managers were selected as key informants because they are responsible for the overall management of the TB, HIV/AIDS, or (integrated) TB-HIV/AIDS program activities in their areas of jurisdiction.

### Group discussions and individual interviews

Open- and closed-ended questions were used both during the group discussions with lay counselors and DOT supporters and the semistructured interviews with program managers. The open-ended question format provides a mechanism through which respondents can use their own words to express their ideas. Such questions are designed to solicit rich, detailed descriptions that are most appropriate for understanding complex issues or processes [26]. Closed-ended questions were used to obtain information on both groups of respondents' demographic details, while open-ended questions gathered information on the factors deterring and facilitating TB patients' acceptance of HCT. Two open-ended questions, which were then elaborated on, formed the starting points of the data-gathering processes: 'In your view, what are the major factors deterring TB patients from undergoing [HCT]?' and 'In your view, what are the major factors encouraging TB patients to undergo [HCT]?'

The face validity (*i.e*., whether the questions make sense as a measure of a construct in the judgment of others) and practicality (*i.e*., likelihood to be successfully understood) of the two open-ended questions were pretested prior to the fieldwork. Managers, DOT supporters, and lay counselors from a district (*i.e*., Motheo) outside of the study area participated in this exercise. The questions were found to be meaningful and valuable in answering the research question.

A total of 32 group discussions included 2 to 3 lay counselors (21 discussions) and 5 to 12 DOT supporters (11 discussions) at a time. Each group interview was conducted in the participants' home language (*i.e*., Sesotho) and lasted approximately one hour.

Respondents were asked for their permission to use an audio recorder. Focus group discussions were moderated by a facilitator, while another research team member took notes to supplement information collected on the audiotapes. Facilitators were trained on how to guide a group discussion and were conversant in the local languages (*i.e*., Sesotho and isiXhosa). Participants were assured about the confidential nature of the discussions and encouraged to express their opinions openly.

The individual interviews with the program managers were conducted by two researchers in either English or Afrikaans. With the consent of interviewees, audio recorders were used. The discussions were facilitated by one researcher/interviewer while another took notes to supplement information collected on the audiotapes. Discussions were confidential and participants were encouraged to express themselves openly and honestly.

### Data analysis

Thematic analysis by means of open-coding has been used in a previous South African study on HIV testing and disclosure among TB patients [27]. In the current study, the information gathered in the group and individual interviews was transcribed verbatim. Data were subjected to recurrent thematic analysis [28]. Two researchers and three research assistants conversant in both Sesotho and English performed thematic analysis by reading and rereading all the transcripts and developing a detailed list of participants' comments in the two areas addressed by the interview questions (*i.e*., views on the facilitators of and barriers to uptake of HIV testing by TB patients). Researchers compared and cross-referenced every identified response to ensure that all respondents' issues, concerns, and ideas were included and to identify common themes. The team met several times to discuss and reassess the overall themes.

## Results

The themes emerging from the focus group discussions and interviews included four main barriers: (1) fears of HIV/AIDS, TB-HIV coinfection, death, and stigma; (2) perceived lack of confidentiality of HIV test results; (3) staff shortages and high workload; and (4) poor infrastructure to encourage, monitor, and deliver HCT. The four main facilitating factors emerging from the group and individual interviews were (1) encouragement and motivation by health workers; (2) alleviation of health worker shortages; (3) improved HCT training of professional and lay health workers; and (4) community outreach activities.

### Fears of HIV/AIDS, TB-HIV coinfection, death, and stigma

The community health workers identified fears of HIV/AIDS, TB-HIV coinfection, and/or death as the most important barrier to HCT acceptance among TB patients:

TB patients only come to the clinic when they are extremely ill and they don't want to be counseled or spoken to about HIV, so they fear having both diseases.

People are afraid to test because it is said that if a person has TB, they automatically have HIV, and they do not want to know.

They are afraid of the fact that HIV is not curable. So when they have TB they are afraid to go and test and hear bad news.

Another prominent barrier to TB patients' acceptance of HCT mentioned by community health workers was fear of experiencing HIV-related stigma and/or discrimination if they tested positive:

When people are ill they are rejected from the community so people would rather not test.

They are afraid of what people will say about them - the stigma associated with AIDS.

People think that HIV/AIDS is a punishment and a shame, so we try to encourage them otherwise.

Among the barriers identified by program managers, the perceived negative emotional experience of a TB patient testing HIV-positive also featured prominently. In fact, all the program manager respondents mentioned patients' fear of being the recipient of HIV-related stigma as a barrier to acceptance of HCT:

They fear stigma in the community.

They fear stigmatization by other patients.

They worry about dual-stigmatization of TB and HIV.

Already the patient is stigmatized, because in our community there are those people who don't accept TB. So patients are already reluctant to have another stigma of HIV, and they just don't go for testing.

All the other patients know that you are going to be tested. Even though it's not a fact that you are going to be positive, others think that you are.

### Perceived lack of confidentiality

Both the community health workers and program managers also perceived that patients were reluctant to accept HCT because they did not trust the healthcare facilities to maintain the confidentiality of their HIV test information:

Patients still do not trust that their results are strictly confidential.

They also say that there is no confidentiality when it comes to HIV.

People say that the nurses and the lay counselors gossip a lot.

In some clinics you find that patients come from the community around the clinic and the people who are doing the counseling are lay counselors, they are community people, the patients know them... they live with them. The patients will not come to that particular facility or they will not agree to test but would rather go somewhere else to test. So there are issues of trust and confidentiality.

Confidentiality plays a big role. Clinics are not really TB and HIV friendly. One person handles a patient and a rapport develops. Then the patient is sent to someone else for [HCT]. They don't feel comfortable with that. They don't want to be sent to someone else.

### Staff shortages and high workload

The community health workers raised pertinent concerns about staff shortages in health facilities and the negative effect this had on uptake of HIV testing by TB patients:

There is a great shortage of nurses, so if they could be increased they would be able to help all patients and not have to send some home.

Similar to the views expressed by community health workers, program managers also identified several delivery-level barriers that played a role in relatively low acceptance rates of HCT services among TB patients. Specifically, program managers noted the lack of appropriately trained staff members, high workloads, and time constraints experienced by professional and lay health workers:

They are suffering in the clinics. There are only a few professional nurses that have to do all the programs. This is a big, big, big concern.

### Poor infrastructure to encourage, monitor, and deliver HCT

Both the community health workers and the program managers often referred to infrastructural problems when encouraging and monitoring HCT services. For example, community health workers were concerned about a lack of information, education, and communication materials provided in local languages, as well as concern about limited access to antiretroviral treatment:

Posters that are in English are not easy to understand as it is not a mother tongue to all.

Some patients say if they test and find out that they are HIV-positive, they will have to be put on the long waiting list for [antiretrovirals] and they will die before they even get help.

The program managers also pointed to a lack of appropriately trained staff members, as well as poor infrastructure to monitor and deliver HCT, as factors contributing to low acceptance rates among TB patients. For example, many clinics did not have systems in place for record-keeping, referral, and patient follow-up for coinfected patients:

The recording is a problem. I remember at some stage I had a problem where I wanted to look at their statistics and all that, and I started to talk to them and asked them where the figures are, but... patients are tested and it is not recorded. There is no system in between patients who have been seen in the TB room that have been transferred to the [HCT] room. The counselors are not recording the information.

### Encouragement and motivation by health workers

The most common suggestion for increasing acceptance of HCT by community health workers was to encourage and motivate TB patients:

We [lay counselors] should tell them that if they've got TB it's vital for them to go test because nowadays TB is never the only problem. Most of them do go and test, but some are still not ready and some lie and say they have tested when they didn't.

We cannot force patients, but we should keep on encouraging them.

Community health workers suggested that both community and professional health workers should engage, or engage more often and more intensely, with patients about their fears of testing HIV-positive, TB-HIV coinfection, and death. The community health workers also suggested that messages to encourage TB patients to accept HCT should be delivered and reinforced by doctors and nurses in order to be optimally effective:

When patients have been seen by doctors, they go more willingly to the clinic to test.

More patients cooperate with nurses. Nurses should talk to them and make them realize the importance of testing for HIV. Nurses should do it because patients respect them and listen to them because they are qualified and they know what they are talking about.

Similar to the community health workers' emphasis on encouragement and motivation, the major proposed facilitator of HCT acceptance among TB patients, as perceived by program managers, was that health workers should follow a patient-centered approach. Such an approach should be characterized by strong confidentiality protection, emotional support, and cultural sensitivity, as well as efforts to understand and acknowledge the cultural beliefs of patients from different backgrounds. This, the program managers suggested, was required to build the strong, provider-patient relationships necessary to increase patient acceptance of HCT:

Patients who did not test the first time they were offered HCT should be continuously advised to do so.

### Alleviation of health worker shortages

The second most prominent theme in community health workers' responses to the question about what would facilitate TB patients' acceptance of HCT was related to the delivery of such services. Specifically, community healthcare workers suggested that increasing the number of health service professionals, particularly those conversant in local languages, would help increase TB patients' acceptance of HCT services:

The doctors here are Nigerian, all three of them. So that also causes a language barrier, because when the patient goes to see the doctor I must go too, and now that makes the patient uncomfortable. If only we could get doctors who know our home language.

There is only one doctor and he only comes on Thursdays, and is always too busy. If there were more doctors it would make a huge difference.

Likewise, the second most prominent factor suggested by the program managers to influence acceptance of HCT among TB patients concerned the lack of available healthcare delivery personnel and professionals. Suggestions to alleviate this problem included increasing the number of healthcare facility staff, improving training for professional and lay health workers, and integrating TB and HIV/AIDS services:

There are a high number of programs in relation to the number of nurses.

The clinics in general are inundated with clients with consequent queuing.

Counseling should include referral of patients to nurses for further counseling about related diseases.

They should strengthen the health system so that patients are treated holistically rather than by specialized personnel in specific programs [*e.g*., nurses trained in the antiretroviral treatment program].

Integrated service provision facilitates uptake of [HCT].

### Improved HCT training of professional and lay health workers

Improved HCT-related training of both nurses and lay counselors, but especially the latter, was the third most prominent theme raised by the program managers in response to the question of how TB patients' uptake of HCT services could be improved:

Improve the quality of training on TB and HIV that professional nurses receive.

There should be ongoing training of lay counselors and DOT supporters on TB and HIV.

Lay counselors should receive comprehensive training.

We've got to improve the skills of lay counselors.

The quality of information imparted by lay counselors should really be improved.

### Community outreach activities

Another prominent factor mentioned by the community health workers was that acceptance of HCT by TB patients should be encouraged not only by healthcare professionals in delivery settings but also through outreach and community activities. There was a strong sentiment in the discussions that lay counselors were able and willing to conduct community outreach:

They should help us do door-to-door [campaigns] and test patients outside of the clinic.

We should be involved in community activities and go talk at churches.

We could have meetings with the community every now and then to talk about these issues.

It would be better if at churches TB and HIV were spoken about.

## Discussion

There is an urgent need in South Africa to increase TB patients' acceptance of HCT services in order to improve patient health outcomes [12,16,27,29,30]. The present study sought to understand patient- and delivery-level factors that influence acceptance of HCT services among TB patients in Free State Province, South Africa from the perspective of two important yet relatively neglected healthcare service stakeholder groups: community health workers and TB and HIV program managers.

Findings from our qualitative study revealed several multilevel barriers to TB patients' acceptance of HCT services. Indeed, both groups of respondents identified several patient-level factors that appeared to reduce TB patients' acceptance of HCT services, including fear of HIV diagnosis and fear of experiencing HIV-related stigma. These patient-level factors hindering HCT uptake have also been identified in previous studies in South Africa [27], Nigeria [31], Burkina Faso [10], and the United Kingdom [9].

Fear of stigmatization as a reason for TB patients' nonuptake of HIV testing also featured prominently in the findings of a qualitative study in Durban, South Africa by Daftary *et al*. in 2007 [27]. This study highlighted TB patients' experiences and perceptions of stigma and disclosure and distinguished between felt and enacted stigma. While the latter concerns the actual experience of a prejudicial act, the former relates to the fear of being discriminated against. It was found that for TB patients unaware of their HIV status, "felt stigma of HIV/AIDS was a critical disincentive for VCT-they could suffer a potential double stigma with an HIV-positive result [[27], p. 574]."

In the current study, both groups of respondents also identified several delivery-level factors that appeared to reduce TB patients' acceptance of HCT services, including lack of trust in staff maintaining the confidentiality of their HIV test results, lack of appropriately trained healthcare personnel, limited availability of antiretroviral medications, poor monitoring of patient care, and fragmented delivery of care services.

In 2000, observations were made that the traditional trust of the community in the health professions was declining in South Africa [[32], pp. 107-108], "although this often appears to be based on expectations of what would happen or on the experience of others rather on individuals' own experience." Lack of patient trust in staff to maintain HIV test confidentiality has also been found in a qualitative study in three clinics with relatively well-established VCT programs in Cape Town, South Africa [33]. Lack of trust and lack of confidentiality in VCT/HCT facilities have also been recorded in a recent attitude survey among clients/patients at three facilities in Pretoria, South Africa to determine whether access to counseling could play a role in improving uptake of VCT [34]. The survey found that lay counsellors felt that they were not adequately trained to do HIV counseling, that they were seeing more clients per day, that time constraints did not allow them to spend enough time with patients during counseling, and that they did not have opportunity to attend debriefing sessions or refresher courses.

Lack of appropriately trained healthcare personnel to service primary healthcare clinics in South Africa [35,36] and in countries with a high burden of TB [37] have also been widely recorded. As Daviaud and Chopra [[35], p. 46] noted in a 2008 study of 340 clinics in six of the poorest districts across four of the nine provinces: "The number of doctors was only 7% of that required, and while the total number of professional nurses was 94% of requirement, there was considerable variation across facilities and districts. The adequacy of provision of enrolled nurses and nursing assistants was worse, at 60% and 17%, respectively."

The theme, poor infrastructure to encourage, monitor, and deliver HCT, recurred in both the focus group discussions with community health workers and the interviews with program managers. Already in 2005, Colvin [[38], p336] assessed the impact of AIDS in terms of a healthcare burden in South Africa negatively, stating that it is unlikely that the public health sector will be able to sustain the increasing costs of treating HIV-positive patients, which means that some form of rationing is inevitable.

Despite studies showing that integration of TB and HIV/AIDS programs may have many benefits for the programs, services, and patients, there are several constraints that undermine the integration process [16]. Lack of integration between the TB and HIV/AIDS programs in sub-Saharan Africa [39] and South Africa [40] continues and TB and HIV/AIDS services essentially remain separate vertical programs.

In addition, community health workers and program managers identified several multilevel facilitators to TB patients' acceptance of HCT services. At the patient level, both groups emphasized taking a patient-centered approach to motivate and encourage acceptance of HCT services. Recommendations were made to healthcare providers to use a "provider-encouragement" approach, whereby health professionals provide continued motivation and support to TB patients to accept HCT services at subsequent visits if they initially declined. At the delivery level, community health workers and program managers suggested providing additional staff resources and personnel (*e.g*., doctors and nurses conversant in local languages, lay counselors to conduct community outreach) as ways to increase HCT acceptance rates.

Summarily, the main factors thought to hinder TB patients from going for HCT were fear of stigmatization, lack of infrastructure, and the unavailability and high workload of healthcare workers. Most of the patient-related factors that the managers perceived to contribute to low uptake of HCT among TB patients-fear, denial, lack of trust and confidentiality, inadequate knowledge-seem closely connected with fear of stigmatization. The managers' responses that link with these factors made it clear that stigmatization is felt on a number of levels: individual, family, community, programmatic, and societal.

Interestingly, there is a large degree of similarity between the barrier and facilitator factors identified by community health workers and program managers in the current study and factors identified in our previous studies among TB patients (being treated in the same setting) [3] and primary healthcare nurses (practicing in the same setting) [14]. The most important barrier factors mentioned by TB patients also included fear. The patients said they were afraid of the HIV test itself (*i.e*., getting blood taken), HIV-related stigma, and consequences of testing HIV-positive: "afraid of people gossiping" and "fear of [side effects] of HIV treatment [11]." When TB patients were asked to suggest what healthcare workers could do to facilitate HCT by TB patients, the most frequent suggestions were to provide them with information about the link between TB and HIV and to motivate and support them emotionally.

In our previous work, primary healthcare nurses most frequently referred to patient-related issues as the main reasons for refusal of HCT by TB patients [14]. Amongst these reasons, the stigma surrounding HIV, patients not wanting to be counseled by lay counselors, denying/fearing that they may have HIV, and preferring to first cope with TB and then deal with HIV featured most prominently. Numerous facility-related barriers were also perceived by the nurses, all relating in some way to lack of sufficient human resources or infrastructural capacity at primary healthcare facilities to provide easily accessible, confidential HCT services. However, despite the existence of a variety of factors discouraging TB patients from going for HCT, there were also numerous positive factors that enabled patients to opt for this service. The main factors viewed by the interviewed nurses to encourage TB patients to take up HCT related to the facilities, staff, and availability of treatment and support. The provision of health education to patients was most often mentioned as a facilitating factor. The second most cited factor was the availability of antiretroviral therapy. However, as shown by Jacobs *et al*., the scale-up of antiretroviral therapy services in South Africa is subject to substantial rationing. These authors observed that the consequences of rationing manifested itself in the high number of patients lost to the system [39].

The present study has several limitations that should be noted. First, results were based solely on respondents' subjective perceptions of barriers and facilitators. One way of counteracting this phenomenon is to involve more than one type of respondent and compare responses across groups, an approach that was applied to data analysis in the present study. A second limitation of this study is that, although the two districts representing the study areas were randomly selected, the inclusion of only four subdistricts limits the generalization of results to the Free State Province. However, the urban-rural mix of selected subdistricts increases the potential generalizability of these findings across both rural and urban settings. Finally, given the exploratory, qualitative nature of the study, causal inferences cannot be inferred. Future empirical research is thus needed to assess the relationship between patient- and delivery-level factors on HCT acceptance rates and to develop multilevel strategies to improve the acceptance of HCT services in care settings.

## Conclusions

Findings from the present study provide important implications for improving patient acceptance of HCT services. Our study also expands on current literature by assessing community health workers and program managers' perspectives on patient- and delivery-level factors that facilitate or impede the acceptance of HCT services among TB patients in Free State Province, South Africa. Suggestions for improving HCT acceptance rates include addressing several patient- and delivery-level factors, such as HIV-related stigma and strengthening of human resources aspects of the healthcare system. Findings from this study have implications for future research needed to identify optimal modes of delivery of health programs and services, with implications not only for patient acceptance and participation rates but also for the adoption, implementation, and sustainability of such programs by healthcare teams, including community health workers and program managers.

## Competing interests

The authors declare that they have no competing interests.

## Authors' contributions

JCH conceived the idea for this work, obtained funding to support it, and wrote the initial and final draft. EW, MCE, NGK, AS, and CR contributed to reframing and reanalysis to produce an improved version. WEN contributed more pertinent implementation science foci. JCH, EW, and WEN formulated the final draft that was contributed to and approved by all authors.
